# A Comprehensive Comparative Study of Direct Vision Internal Urethrotomy and Urethroplasty in Short-Segment Bulbar Urethral Strictures

**DOI:** 10.7759/cureus.76567

**Published:** 2024-12-29

**Authors:** Gaurav Babelay, Rohit Upadhyay, Ahsan Ahmad, Nikhil Ranjan, Kumar Dheeraj

**Affiliations:** 1 Department of Urology, Indira Gandhi Institute of Medical Sciences, Patna, IND

**Keywords:** bulbar urethral stricture, direct vision internal urethrotomy, urethral stricture, urethroplasty, urethrotomy

## Abstract

Background

Currently, there is no data on the prevalence of urethral stricture illness in India. For short-segment bulbar urethral stricture, end-to-end anastomosis is the gold standard of care. The purpose of this study was to find where the direct vision internal urethrotomy (DVIU) exists in today's era. Also, it compared DVIUs with urethroplasty. Further, the comparison was performed in the urethroplasty group which was converted into two sub-groups; buccal mucosal graft (BMG) and anastomotic urethroplasty.

Materials and methods

It was a randomized prospective interventional study. The study was conducted at the Department of Urology at Indira Gandhi Institute of Medical Sciences (IGIMS), Patna, India. The total duration of the study was one year and six months. Ethical approval for the conduction of the study has been obtained from the institutional ethics committee (IEC) of IGIMS, Patna, Bihar, India under letter number 840/IEC/IGIMS/2022 dated 10 December 2022.

Results

The study included two comparisons, one between urethrotomy and urethroplasty, that found significant differences in the International Prostate Symptom Score (IPSS) scores at three months. However, the IPSS scores were found to be insignificant between the groups at six months. Also, no statistically significant difference was observed in the International Prostate Symptom Score-Quality-of-Life (IPSS-QOL) between the two groups at three and six months. The statistically significant difference between them was observed in the maximum urinary flow rate (Qmax) and the International Index of Erectile Function-5 (IIEF-5) scores at three and six months, respectively. Another comparison was done between BMG and the excision and primary anastomosis (EPA) groups, where there was no statistical difference observed between the groups in terms of IPSS, IPSS-QOL, Qmax, and recurrence at three and six months. However, there was a statistical difference observed in IIEF-5 scores between the groups at three and six months, respectively. The mode of anaesthesia in the DVIU group was either total intravenous anaesthesia (TIVA) or spinal anaesthesia. On the other hand, all cases of BMG urethroplasty required general anaesthesia with nasal intubation and all cases of EPA required spinal anaesthesia.

Conclusion

It has been concluded that in today’s era, DVIU can be considered for de-novo short-segment bulbar urethral stricture in individuals who are concerned about sexual life. And, for short-segment bulbar urethral stricture less than 2 cm, BMG is a better alternative to EPA as it is associated with less erectile dysfunction. A decrease in erectile function is more common in anastomotic urethroplasty as compared to BMG urethroplasty. Further studies comparing muscle sparing, nerve sparing, and vessel sparing are required to address this problem.

## Introduction

The incidence of urethral stricture illness in developed nations ranges from 0.6% to 0.9% [1,2], and it significantly impacts the quality of life of patients [2]. However, no prevalence of urethral stricture disease in India has been reported yet [3]. The aetiology of urethral strictures varies from country to country across the world. Among all causes, iatrogenic and idiopathic strictures are common in the Indian population [4].

Stricture disease tends to have an intense impact on quality of life. Possible complications include urinary tract infections (UTIs), bladder calculi, urethra-cutaneous fistulas, sepsis, and renal failure. Numerous therapeutic approaches have been developed to treat these patients, but none are effective for all types of strictures. The stricture's position, length, depth, and density must be identified to create a suitable treatment strategy. Nowadays, there are many closed and open methods for treating urethral stricture disorders, but no method has been yet proven to be universal [4-6].

Optical internal urethrotomy and dilatation are the most often utilized initial techniques for treating solitary bulbar urethral strictures that are short (less than 1.5 cm) [5,6]. Nevertheless, these treatments have a 39% to 73% recurrence-free rate; repeated urethrotomies or dilatations have even lower success rates and are therefore not cost-effective [7-10].

However, buccal mucosal graft (BMG) urethroplasty is the preferred treatment for anterior urethral strictures greater than 2 cm [11-14]. The most effective treatment for short-segment bulbar urethral stricture is end-to-end anastomosis [15,16]. Concerns that urethral transection could negatively impact the urethral blood flow, leading to urethral ischemia and sexual side effects, have grown recently [17-20]. The purpose of this study was to find whether the direct vision internal urethrotomy (DIVU) exists in today's era.

The aim of conducting this study was to assess the outcomes of urethrotomy and urethroplasty as well as a comparison of anastomotic and augmented BMG urethroplasty in short-segment bulbar urethral stricture.

## Materials and methods

Study design

The study was a randomized prospective interventional study. The study was conducted at the Department of Urology at Indira Gandhi Institute of Medical Sciences (IGIMS), Patna, India. The total duration of the study was one year and six months.

Study population

A total of 40 patients were enrolled in this study. The inclusion criteria for enrolled patients were patients with short-segment bulbar urethral stricture less than 2 cm, patients aged between 18 and 50 years of age, participants who completed follow-up at three and six months, and participants who signed informed consent. The exclusion criteria were participants with end-stage renal disease (ESRD), balanitis xerotica obliterans (BXO), UTIs, pelvic fracture urethral injury (PFUI), malignant stricture, uncorrected coagulopathy, abnormal bladder, and recurrent stricture. The participants were further divided into 1:1 ratios between the two groups, respectively.

Group A included 20 patients who underwent DVIU, and Group B included 20 patients who underwent urethroplasty. Group B was further subdivided into two subgroups: patients who underwent BMG and patients who underwent anastomotic urethroplasty. Both these subgroups had 10 patients each.

Study procedure

DVIU procedure was described by Sachse, in which the procedure includes a single cut made at 12 o’clock position in the scar tissue, till the scar is incised completely. And, in the urethroplasty procedure, circumferential mobilization of the bulbar urethra was done. The urethra was transacted at the level of block and all fibrotic tissue was removed till a supple urethra was obtained proximally and distally. Spatulation of the distal urethra dorsally and proximal urethra ventrally was done. The segments were anastomosed over a 14 or 16F catheter using 4/0 Vicryl (Ethicon Inc., Somerville, USA) sutures with six stitches at 1, 11, 3, 6, 5, and 7 o’clock positions.

Then, in the BMG urethroplasty procedure, circumferential mobilization of the bulbar urethra was done. The urethra was incised 1 cm proximal and distal to the level of maximum obliteration till a supple urethra was obtained at both ends. Spatulation of both distal and proximal urethra was done dorsally. The buccal mucosal graft was harvested from the left or right cheek depending upon the patient's preference and the condition of the buccal mucosa. The graft was fixed dorsal to the corpus cavernosa. The urethra was anastomosed to the mucosa on the left and the spongiosum on the right. The suture material used was Vicryl 4/0. A 14F or 16F silicone catheter was used to drain the bladder. Postoperatively, the urethral catheter was removed after five days in DVIU and after three weeks in the urethroplasty group. All patients were followed up postoperatively at three and six months, respectively.

Data collection

Patients underwent detailed history taking and clinical examination and were subjected to the following baseline investigations as urine routine examination, complete blood count (CBC), prothrombin time-international normalized ratio (PT-INR), kidney function test (KFT), random blood sugar (RBS), ultrasound-abdomen (USG abdomen), International Prostate Symptom Score (IPSS), and International Index of Erectile Function-5 (IIEF-5) score. Specific investigations included retrograde urethrogram-micturating cystourethrogram (RGU MCU), uroflowmetry, and cystourethroscopy.

Statistical analysis

Data was entered initially in Microsoft Excel (Microsoft® Corp., Redmond, WA, USA) for categorization. Data analysis was performed using the Statistical Package for the Social Sciences (IBM SPSS Statistics for Windows, IBM Corp., Version 24.0, Armonk, NY). Normal distribution of the data between both groups was followed. Data contained both continuous and categorical variables. Quantitative variables were expressed as mean ± standard deviation (SD) or n (%) or n. Unpaired t-tests and chi-square tests were used to obtain the t-value and p-value. A p-value was considered significant at <0.05. The logistic regression model was used to obtain the odds ratio (OR).

Ethical clearance

Ethical approval has been obtained from the Institutional Ethics Committee (IEC) of IGIMS under letter number 840/IEC/IGIMS/2022 dated 10 December 2022.

## Results

Table 1 represents the patients' demographics. The mean age group of the DVIU group was 32.25±10.3 while the mean age in the urethroplasty group was 32.05±10.44. The most common aetiology of stricture in both groups was idiopathic. It was followed by iatrogenic and inflammatory in the DVIU group whereas in the urethroplasty group, inflammatory was the second most common cause, followed by iatrogenic. The mean length was 1.41±0.3cm in the DVIU group and 1.59±0.33cm in the urethroplasty group. The most common location of stricture in both the groups was distal followed by proximal and mid-bulbar urethra.

**Table 1:** Patient demographics Data was presented as either mean±SD or n (%). A p-value was considered significant at <0.05. An unpaired t-test or chi-square test was used to obtain a p-value. DVIU: direct vision internal urethrotomy

Parameters	DVIU (n=20)	Urethroplasty (n=20)	p-value
Age (in years)	32.25±10.3	32.05±10.4	0.95
Stricture aetiology			
Idiopathic	12 (60%)	10 (50%)	0.584
Inflammatory	03 (15%)	08 (40%)	
Iatrogenic	05 (25%)	02 (10%)	
Length of the bulbar urethra	1.41±0.3	1.59±0.3	0.06
Location of the bulbar urethra			
Proximal bulbar	07 (35%)	05 (25%)	0.6
Mid bulbar	02 (10%)	04 (20%)	
Distal bulbar	11 (55%)	11 (55%)	

Table 2 represents a comparison of parameters between DVIU and urethroplasty groups, respectively. There was a statistically significant difference in the IPSS score between the two groups at three months; however, the IPSS score difference was insignificant at six months. And, there was no statistically significant difference in the International Prostate Symptom Score-Quality-of-Life (IPSS-QOL) between the two groups at three as well as six months. In the urethroplasty group, the post-operative maximum urinary flow rate (Qmax) improved from 7.9±4.05 to 26±2.9 after three months and changed to 23.44±2.5 after six months.

**Table 2:** Comparison of parameters between DVIU and urethroplasty groups Data was presented as mean±SD. A p-value was considered significant at <0.05. An unpaired t-test was used to obtain a p-value. DVIU: direct vision internal urethrotomy; IPSS: International Prostate Symptom Score; IPSS-QOL: International Prostate Symptom Score-Quality-of-Life; Qmax: maximum urinary flow rate; IIEF-5: International Index of Erectile Function-5

Parameters	DVIU (n=20)	Urethroplasty (n=20)	p-value
IPSS (Pre-op)	22.75±3.48	24.5±4.3	0.16
IPSS (at 3 months)	9.4±4.4	6.3±2.2	0.01
IPSS (at 6 months)	9.54±3.71	7.67±2.06	0.08
IPSS-QOL (Pre-op)	4.8±0.89	5.05±0.89	0.34
IPSS-QOL (at 3 months)	0.83±0.99	0.55±0.69	0.45
IPSS-QOL (at 6 months)	0.87±0.99	0.6±0.6	0.43
Qmax (Pre-op)	8.3±3.1	7.9±4.05	0.76
Qmax (at 3 months)	21.2±3.76	26±2.9	0.001
Qmax (at 6 months)	20±3.5	23.4±2.5	0.004
IIEF-5 Score (Pre-op)	20.3±2.6	18.5±15.2	0.13
IIEF-5 Score (at 3 months)	19.4±2.4	15.2±4.1	0.003
IIEF-5 Score (at 6 months)	20.4±2.4	17±4.2	0.017

Recurrence in the DVIU group was 35%. Of these, 10% of strictures recurred within three months of surgery while 25% recurred between three and six months of surgery. Recurrence in the urethroplasty group was 10% all of which recurred between three and six months of surgery. The OR was 4.84 in recurrence among the participants. Table 3 depicts the recurrence among the participants.

**Table 3:** Recurrence among the participants in groups Data was presented as n/n (%). A logistic regression model was used to obtain the odds ratio. DVIU: direct vision internal urethrotomy

	Overall recurrence	Within 3 months	Within 3-6 months	Odds ratio
DVIU	7/20 (35%)	2/20 (10%)	5/20 (25%)	4.84
Urethroplasty	2/20 (10%)	0/20	2/20	

The mode of anaesthesia in the DVIU group was either total intravenous anaesthesia (TIVA) or spinal anaesthesia. On the other hand, all cases of BMG urethroplasty required general anaesthesia with nasal intubation and all cases of excision and primary anastomosis (EPA) required spinal anaesthesia. The mean haemoglobin drop in the DVIU group was 0.73±0.23 g/dL whereas in the urethroplasty group, it was 1.2±0.3 g/dL. Table 4 represents intraoperative and postoperative outcomes among participants.

**Table 4:** Intraoperative and postoperative outcomes Data was presented as either mean±SD or n (%). A p-value was considered significant at <0.05. Unpaired t-test and chi-square test were used to obtain the p-value. DVIU: direct vision internal urethrotomy; TIVA: total intravenous anaesthesia; SA: spinal anaesthesia; GA: general anaesthesia

Outcomes	DVIU (n=20)		Urethroplasty (n=20)	t-value/chi-square value	p-value
Mode of anaesthesia	TIVA-12; SA-8		GA-10; SA-10	-	-
Hb drop (gm/dL)	0.73±0.23		1.2±0.3	5.56	0.001
Hospital stays (in days)	2.4±0.59		4.25±1.97	4.02	0.001
Clavin-Dindo complication					
Grade I	14		11	1.64	0.43
Grade II	6		8		
Grade III	0		1		

The pre-op IPSS score in the EPA group was 26.4±4.3 which changed to 6.5±2.23 after three months and 7.56±2.4 after six months. There was no statistically significant difference in the IPSS score at three and six months between the two groups. No statistically significant improvement in IPSS-QOL was observed at three and six months between the two groups. Table 5 shows the comparison of various parameters between BMG and EPA groups, respectively.

**Table 5:** Comparison of parameters between BMG and EPA groups Data was presented as mean±SD. A p-value was considered significant at <0.05. An unpaired t-test was used to obtain the p-value. IPSS: International Prostate Symptom Score; IPSS-QOL: International Prostate Symptom Score-Quality-of-Life; Qmax: maximum urinary flow rate; IIEF-5: International Index of Erectile Function-5; BMG: buccal mucosa graft; EPA: excision and primary anastomosis

Parameters	BMG (n=10)	EPA (n=10)	t-value	p-value
IPSS (Pre-op)	22.6±3.5	26.4±4.3	2.16	0.04
IPSS (at 3 months)	6.2±2.3	6.5±2.2	0.29	0.77
IPSS (at 6 months)	7.77±1.78	7.5±2.4	-0.28	0.82
IPSS-QOL (Pre-op)	4.7±0.8	5.4±0.8	1.95	0.06
IPSS-QOL (at 3 months)	0.5±0.7	0.6±0.6	0.34	0.73
IPSS-QOL (at 6 months)	0.5±0.7	0.5±0.7	0.00	0.8
Qmax (Pre-op)	7.7±4.8	8.2±2.3	0.29	0.86
Qmax (at 3 months)	26.7±3.09	25.7±2.7	-0.77	0.45
Qmax (at 6 months)	23.5±2.7	23.3±2.34	-0.17	0.85
IIEF-5 Score (Pre-op)	20±2.1	17.7±4.1	-1.57	0.23
IIEF-5 Score (at 3 months)	18±3.4	13.6±3.7	-2.76	0.03
IIEF-5 Score (at 6 months)	20.1±2.9	15.1±4.04	-3.17	0.01

## Discussion

This study was conducted to analyze the outcome of DVIU and urethroplasty in short-segment bulbar urethral stricture by using multiple parameters. The performance of EPA urethroplasty was compared with BMG urethroplasty to evaluate if BMG urethroplasty can be a better alternative for the management of such patients.

In our study, the cut-off for short-segment bulbar urethral stricture was less than 2 cm with mean age group 32.25 and 32.05 years for DVIU and urethroplasty groups, respectively. A study done by Jahan MS et al. reported a similar cutoff of bulbar urethra showing almost similar age groups of 33.6 and 33.9 in DVIU and urethroplasty with mean stricture lengths of 1.5 and 1.8 cm, respectively [21].

The stricture aetiology in the study by Stormont TJ et al. was primarily iatrogenic (47%), and the average patient age at diagnosis was 64 years (range 10 to 96). In 96% of cases, the strictures were smaller than 2 cm in length [22]. Proximal bulbar urethra was the most frequently reported site. Patients in Beysens M et al.'s study had an average stricture length of 3 cm and an average age of 40 years. Idiopathic and iatrogenic were found to be the most prevalent etiologies [23].

In our study, the mean pre-op IPSS was 22.75 and 24.5 in DVIU and urethroplasty groups, respectively and the comparison was statistically non-significant. This score reduced to 9.4 and 6.3, respectively in the DVIU and urethroplasty groups at three months after surgery and the difference was now statistically significant between these two groups. In the second arm of this study, the mean pre-op IPSS was 22.26 and 26.4 in BMG and EPA urethroplasty groups, respectively and the comparison was statistically significant. In the 2016 study by Yuri P et al., voiding symptoms after BMG and EPA were observed in 12.5% (7/56) and 14% (8/57) of patients, respectively; the difference was not statistically significant [24].

In a study by Beysens M et al. in 2021, the mean pre-op IPSS was 23 and 21 in free graft urethroplasty (FGU) and EPA urethroplasty groups, respectively. After six weeks of surgery, IPSS significantly reduced to 10 and 7 in the FGU and EPA urethroplasty groups, respectively [23].

In this study, the mean Qmax pre-op was statistically insignificant between the two groups (DVIU: 8.3 mL/s and urethroplasty: 7.9 mL/s). After six months also Qmax (DVIU: 20 mL/s and urethroplasty: 23.44 mL/s) was more in the urethroplasty group (p=0.004). Similarly, a study by Jahan MS et al. reported that Qmax improvement was greater in the urethroplasty group at three months (DVIU: 18.4 mL/s and urethroplasty: 20.2 mL/s) and at six months (DVIU: 17.8 mL/s and urethroplasty: 19.6 mL/s); both comparisons being clinically significant [21].

Some findings of this study are also supported by Beysens M et al. in which the mean IIEF-5 score decreased in both EPA and FGU at six weeks and was clinically significant for the EPA group (p=0.005). But, after six months, it was no longer significant between the two groups (p=0.313) [23]. Also, the recurrence rate in the DVIU and urethroplasty groups was 35% and 10%, respectively (OR: 4.84). Similarly, in a study by Jahan MS et al., 24% and 28% recurrence rates were reported for DVIU at three and six months, respectively, while in the urethroplasty group, no recurrence was reported at three months and 4% recurrence was reported at six months [21].

The limitations of the study included small sample size, short duration of follow-up, different operating surgeons and that it was a single-centric study.

## Conclusions

The study concluded that as compared with DVIU, urethroplasty has excellent success rates, longevity, low morbidity, and complications in short-segment bulbar urethral stricture. Therefore, presently DVIU can be considered in de-novo short-segment bulbar urethral stricture, following shared decision-making that takes into account the recurrence rate, particularly in individuals concerned about sexual life. For short-segment bulbar urethral strictures that are less than 2 cm, BMG was found to be a better alternative to EPA as it is associated with less erectile dysfunction. A decrease in erectile function was more common in anastomotic urethroplasty as compared to BMG urethroplasty. Recovery in erectile function largely depends upon baseline characteristics. However, further studies comparing muscle sparing, nerve sparing, and vessel sparing are required to address this problem.
